# Increased Apolipoprotein A1 Expression Correlates with Tumor-Associated Neutrophils and T Lymphocytes in Upper Tract Urothelial Carcinoma

**DOI:** 10.3390/cimb46030139

**Published:** 2024-03-07

**Authors:** Chih-Chia Chang, Chia-Bin Chang, Chiung-Ju Chen, Chun-Liang Tung, Chi-Feng Hung, Wei-Hong Lai, Cheng-Huang Shen, Chang-Yu Tsai, Ya-Yan Lai, Ming-Yang Lee, Shu-Fen Wu, Pi-Che Chen

**Affiliations:** 1 Department of Radiation Therapy and Oncology, Ditmanson Medical Foundation Chia-Yi Christian Hospital, Chiayi 600566, Taiwan; 07229@cych.org.tw; 2 Department of Urology, Ditmanson Medical Foundation Chia-Yi Christian Hospital, Chiayi 600566, Taiwan; bining1029@hotmail.com (C.-B.C.); cfhung1017@gmail.com (C.-F.H.); 07458@cych.org.tw (W.-H.L.); 01712@cych.org.tw (C.-H.S.); j59691033@hotmail.com (C.-Y.T.); 3 Department of Laboratory Medicine, Ditmanson Medical Foundation Chia-Yi Christian Hospital, Chiayi 600566, Taiwan; 02125@cych.org.tw; 4 Department of Human Biobank, Ditmanson Medical Foundation Chia-Yi Christian Hospital, Chiayi 600566, Taiwan; 5 Department of Pathology, Ditmanson Medical Foundation Chia-Yi Christian Hospital, Chiayi 600566, Taiwan; cych02112@gmail.com; 6 Department of Biomedical Sciences, Institute of Molecular Biology, National Chung Cheng University, Chiayi 621301, Taiwan; 7 Ditmanson Medical Foundation Chia-Yi Christian Hospital, Chiayi 600566, Taiwan; 01194@cych.org.tw; 8 Department of Hematology and Oncology, Ditmanson Medical Foundation Chia-Yi Christian Hospital, Chiayi 600566, Taiwan; 9 Department of Biomedical Sciences, Epigenomics and Human Disease Research Center, National Chung Cheng University, Minhsiung, Chiayi 621301, Taiwan; biosfw@ccu.edu.tw

**Keywords:** upper tract urothelial carcinoma, tumor-infiltrating neutrophils, tumor-infiltrating T lymphocytes, apolipoprotein A1

## Abstract

An increased neutrophil-to-lymphocyte ratio (NLR) is a poor prognostic biomarker in various types of cancer, because it reflects the inhibition of lymphocytes in the circulation and tumors. In urologic cancers, upper tract urothelial carcinoma (UTUC) is known for its aggressive features and lack of T cell infiltration; however, the association between neutrophils and suppressed T lymphocytes in UTUC is largely unknown. In this study, we examined the relationship between UTUC-derived factors and tumor-associated neutrophils or T lymphocytes. The culture supernatant from UTUC tumor tissue modulated neutrophils to inhibit T cell proliferation. Among the dominant factors secreted by UTUC tumor tissue, apolipoprotein A1 (Apo-A1) exhibited a positive correlation with NLR. Moreover, tumor-infiltrating neutrophils were inversely correlated with tumor-infiltrating T cells. Elevated Apo-A1 levels in UTUC were also inversely associated with the population of tumor-infiltrating T cells. Our findings indicate that elevated Apo-A1 expression in UTUC correlates with tumor-associated neutrophils and T cells. This suggests a potential immunomodulatory effect on neutrophils and T cells within the tumor microenvironment, which may represent therapeutic targets for UTUC treatment.

## 1. Introduction

Upper tract urothelial carcinoma (UTUC) comprises 5–10% of all urothelial carcinomas [1]. Nonetheless, it has a more aggressive clinical behavior than bladder cancer. At initial diagnosis, nearly 60% of UTUCs are invasive (≥pT2) compared with 15–25% of bladder cancers [1]. In Taiwan, the incidence of UTUC is relatively higher due to exposure to aristolochic acid, especially in the southwest area, accounting for 20–25% of all urothelial cancers [2,3]. Risk factors such as tobacco exposure and carcinogenic aromatic amine are shared between UTUC and bladder cancers [4,5]. However, UTUC and bladder cancer exhibit significant differences in the prevalence of genomic alterations. Compared with bladder cancer, UTUC has more clonal mutation members, such as *TP53*, *PIK3CA*, and *FGFR3* [6]. Mismatch repair deficiency, a cause of Lynch syndrome, has recently been reported to be associated with a 9% risk of developing UTUC, compared to 1% for bladder cancer [7]. This suggests that UTUC is the most common urologic cancer associated with Lynch tumors, potentially leading to a wide spectrum of malignancies [7]. Unlike the diverse subtypes found in bladder cancer, UTUC is molecularly classified as a luminal–papillary feature and is characterized by T cell deficiency within the tumor tissue [8].

Neutrophils are the most abundant circulating leukocytes and play a crucial role as first responders to inflammation and infection. Neutrophil-infiltrated tissues contribute to chronic inflammation and can even trigger tumorigenesis [9]. Circulating neutrophils are recruited into tumors and serve as the dominant protumor immune cells in breast cancer [10]. Numerous studies indicate that tumor-associated neutrophils not only promote tumor progression and metastasis but also suppress T lymphocyte activity [11,12,13]. In the peripheral blood, a high neutrophil-to-lymphocyte ratio (NLR) serves as a biomarker for poor prognosis of various cancers, including urothelial carcinoma [12,14]. An elevated NLR reflects the relative depletion of lymphocytes in the circulation and tumor and is associated with a low response rate to immune checkpoint inhibitor therapy [15,16]. Previous studies have examined a correlation between higher NLR values in UTUC patients and worse outcomes [17,18]. In bladder cancer, patients with higher NLR values and tumor-infiltrating neutrophils also tend to exhibit poor overall survival [19], whereas higher tumor-infiltrating lymphocytes (TILs) are associated with longer survival. These studies underscore the interplay between neutrophils and lymphocytes in the tumor microenvironment. Elevated neutrophil levels may be involved in UTUC progression and lymphocyte suppression in the blood [20,21]. Although NLR values may be correlated with clinical outcomes in UTUC, their association with suppressed lymphocytes remains unclear. Furthermore, the relationship between tumor-infiltrating neutrophils and lymphocytes in UTUC is unknown.

Here, we identified the tumor-derived factors that exhibit associations with neutrophils and lymphocytes in UTUC. The secretome obtained from UTUC tumor tissue caused T cell suppression by neutrophils. Apolipoprotein A1 (Apo-A1), a predominantly expressed protein within UTUC tumor tissue, was correlated with the NLR value in the blood. In addition, we assessed tumor-infiltrating neutrophils and T lymphocytes to validate their correlation with Apo-A1.

## 2. Materials and Methods

### 2.1. Study Participants

The study participants were recruited from customary urological practices at the Chia-Yi Christian Hospital. The pathological confirmation of UTUC was performed by customary urological practices, including an endoscopic biopsy and the surgical resection of urinary tract cancers. A computed tomography scan was used to confirm the tumor lesions of the renal pelvis and ureter. These cases included patients presenting hematuria symptoms and diagnosed with UTUC. The cases diagnosed with other malignancies or using immunotherapy drugs met the exclusion criteria. All tumor specimens were collected from UTUC patients undergoing nephroureterectomy. Fresh peripheral blood samples were obtained from patients and healthy donors within 6 h after collection. Serum samples were collected and frozen at −80 °C until they were analyzed. The serum Apo-A1 level was determined in an ADVIA Chemistry XPT System analyzer (Siemens Medical Solutions, Malvern, PA, USA) by the immunoturbidimetric method using commercially available kits (Siemens Healthcare Diagnostics, Berkeley, CA, USA). The assay range for Apo-A1 was 15–260 mg/dL. The hematological samples were processed by the same laboratory, and the data about the neutrophils and lymphocytes in the peripheral blood of the study participants are shown in Table 1. This study was approved by the Ethics Committee of Chia-Yi Christian Hospital (No. 2020121) in Taiwan and followed the Declaration of Helsinki ethical principles for medical research involving human subjects.

### 2.2. Collection of the Supernatant from Primary UTUC Tumor

Fresh primary tumor tissues obtained from surgery were gently rinsed with cold 1× PBS to remove blood and mucus. They were then placed into a collection tube and immersed in cold RPMI1640 medium (HIMEDIA, Mumbai, India) containing 10% fetal bovine serum (FBS, Gibco, Grand Island, NY, USA), 100 unit/mL penicillin (HIMEDIA), and 100 μg/mL streptomycin (HIMEDIA). The tissues were transported on ice to the laboratory and transferred into a culture plate. Subsequently, tissues were minced into small pieces after the removal of the transport medium. Another fresh medium was then used to incubate with the minced tissues (4 mL of medium per gram of tumor). After 24 h, the supernatants were collected after centrifugation.

### 2.3. Treatment of Neutrophil and Co-Culture Experiment

The human whole blood neutrophil isolation kit (Biolegend, San Diego, CA, USA) was used to isolate neutrophils from the peripheral blood of healthy donors. Neutrophils were resuspended with culture medium (10% FBS, 100 unit/mL penicillin, and 100 μg/mL streptomycin in RPMI1640 medium). Prior to neutrophil/T cell co-culture, neutrophils were pre-treated with the 20% or 50% supernatants from UTUC tumor tissue, in a 5% CO_2_ environment at 37 °C. One hour later, neutrophils were washed with 1× PBS twice and then suspended in the culture medium.

CD3 T cells were isolated from peripheral blood mononuclear cells of healthy donors using a human T lymphocyte enrichment kit (BD Bioscience, San Jose, CA, USA). The CD3 T cells were labeled with carboxyfluorescein succinimidyl ester (CFSE, ThermoFisher, Waltham, MA, USA). Then, 1 × 10^5^ neutrophils were co-cultured with CFSE-labeled CD3 T cells, at a ratio of 1:1, in 96-well plates, upon anti-CD3/CD28 stimulation (Dynabeads^TM^ human T activator CD3/CD28, ThermoFisher, Waltham, MA, USA). The percentages of proliferating T cells were analyzed by assessing the CFSE-diluted population on day 4.

### 2.4. Proteomic Array and Analysis

The supernatants from UTUC tumor tissues were analyzed using a Human XL Cytokine Array Kit (R&D Systems, Minneapolis, MN, USA) according to the manufacturer’s protocol. Protein dots were performed by chemiluminescence detection using a MultiGel-21 imaging system (TOPBIO, Taipei, Taiwan). The spot pixel density was quantified using MATLAB software (version R2018a). Differential factors that were upregulated in tumor tissue were identified based on the following criteria: fold change > 2 and *p* value < 0.05, compared to the control medium. The heatmap of differential factors in tumor tissues was made using GraphPad Prism Software, version 8 (GraphPad Software Inc., San Diego, CA, USA).

### 2.5. Isolation of Tumor-Infiltrating Cells

The primary tumor tissue was minced into small pieces and digested with collagenase D and DNase I for 50 min at 37 °C. Cells were isolated by passing the tissue through 100 μm cell strainers (BD Biosciences, San Jose, CA, USA), and they were resuspended with culture medium. The resuspended cells were subjected to Ficoll-Paque gradient centrifugation. The cells harvested from the interface were washed twice with 1× PBS, and the cell numbers were determined after replacing the PBS with culture medium.

### 2.6. Flow Cytometry

The cells were resuspended with staining buffer (1× PBS containing 2% FBS and 2 mM EDTA) and then stained with fluorescent dye-conjugated Ab at 4 °C, for 30 min in the dark. After Abs staining, cells were washed with 1× PBS twice and then analyzed by flow cytometry. The cells from the neutrophil/CD3 T cell co-culture experiment were washed with 1× PBS once before analysis. An Accuri C6 plus flow cytometer (BD Biosciences) was used for evaluating the cell markers, which were analyzed using FlowJo software (version 10). Anti-human CD66b-PE (clone 6/40c), anti-human CD45-PECy7 (2D1), anti-human CD15-APC (SSEA-1), and anti-human CD4-APC (RPA-T4) were purchased from Biolegend (San Diego, CA, USA). Anti-human CD3-PE (UCHT1) and anti-human CD8-FITC (RPA-T8) were purchased from BD Biosciences.

### 2.7. Statistical Analysis

GraphPad Prism Software, version 8 (GraphPad Software Inc.), was used for statistical analysis. Correlations were assessed using nonparametric Spearman correlation. Statistical comparisons were performed using an unpaired *t*-test. *p*-values < 0.05 were considered statistically significant. The characteristics of the study participants are presented as means ± standard deviations.

## 3. Results

### 3.1. UTUC-Derived Factors Modulate Neutrophils to Suppress T Lymphocytes

**Figure 1:**
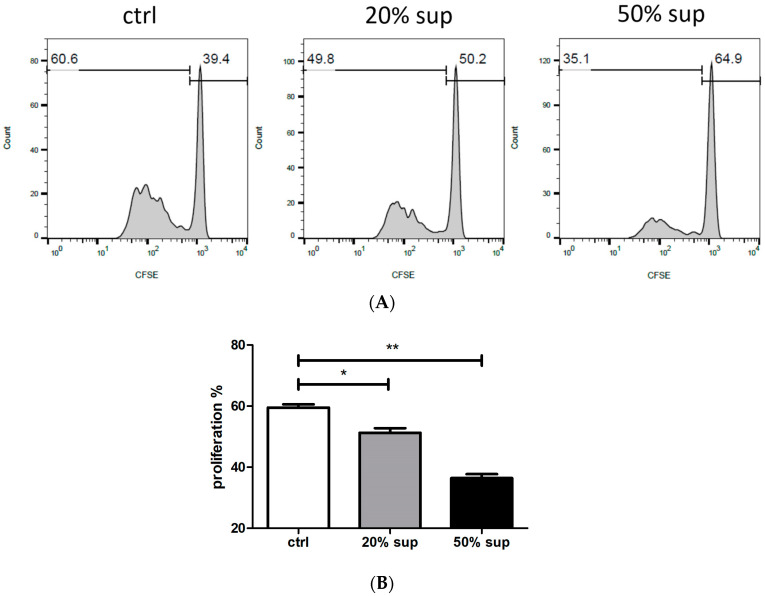
UTUC modulates neutrophils to inhibit T cell proliferation. (**A**) Neutrophils from healthy donors were pretreated with 20% or 50% supernatant (20% sup, 50% sup) obtained from primary UTUC tumor tissue, or pretreated with control medium (ctrl), for 1 h. After washing with PBS, the neutrophils were co-cultured with CFSE-labeled CD3 T cells at a 1:1 ratio. The proliferating cells were analyzed by flow cytometry on day 4. The histograms represent the percentage of T cell proliferation. (**B**) Statistical results for the ctrl, 20% sup, and 50% sup groups are presented. One of the three independent experiments is represented. * *p* < 0.05 and ** *p* < 0.01.

Tumor-secreted factors impact neutrophils, leading to the suppression of T cell immunity in various cancers [22,23,24]. Some studies have suggested that tumor-derived factors CCL20 or GM-CSF induce PD-L1 induction on neutrophils [22,23]. Therefore, we determined whether factors derived from UTUC cause neutrophils to inhibit T cell activity. We collected the supernatants from the primary tumor tissue of UTUC following surgical resection. Peripheral purified neutrophils from healthy donors were treated with 20% or 50% supernatants from UTUC tumor tissue and subsequently co-cultured with T cells. The supernatants pre-incubated with neutrophils reduced T cell proliferation in a dose-dependent manner (Figure 1A,B). This suggests that neutrophils exert inhibitory effects on T cells in UTUC patients. In addition, UTUC patients (*n* = 14) exhibited a higher neutrophil-to-lymphocyte (NLR) ratio compared with healthy subjects (*n* = 20) (Table 1). These results demonstrate a relationship between the modulation of neutrophils and the suppression of T cells in UTUC.

**Figure 2:**
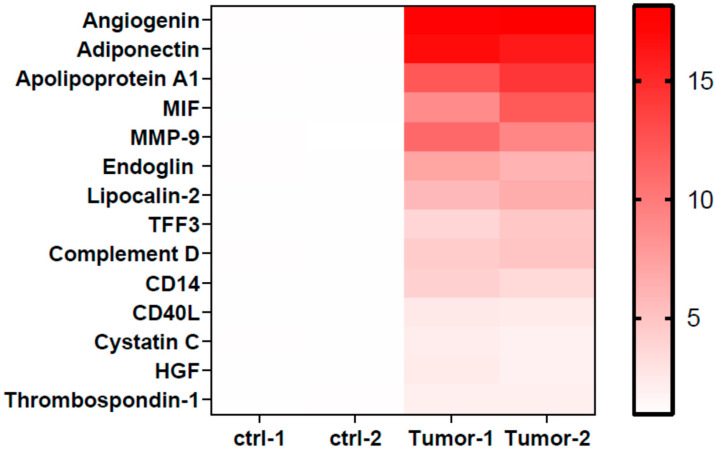
Heatmap of protein level of factors upregulating in tumor tissues. The protein dots data from antibody arrays were quantified, and the relative expression in supernatants of UTUC tumors (*n* = 2) and control mediums (*n* = 2) were further analyzed. The heatmap represents the fold change > 2 in UTUC compared with control (*p* < 0.05).

**Figure 3:**
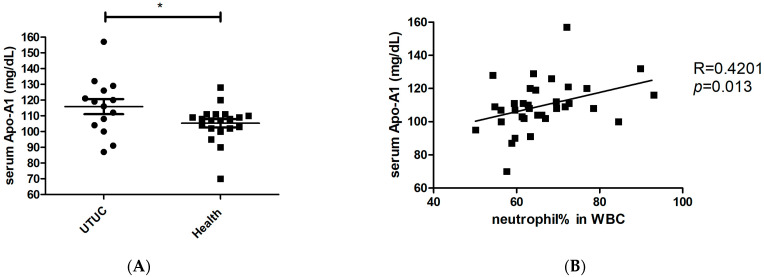

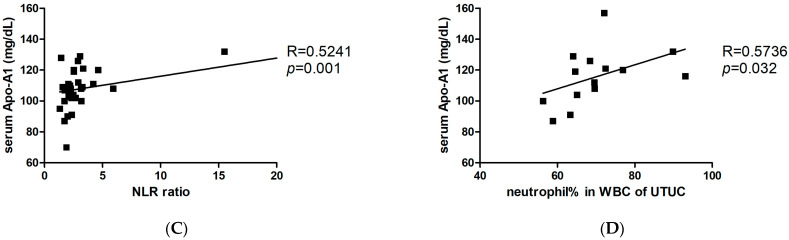
Association between higher neutrophil-to-lymphocyte ratio and increased serum Apo-A1 level in UTUC patient blood. (**A**) Serum Apo-A1 levels (mg/dL) in UTUC patients (*n* = 14) and healthy subjects (*n* = 20). (**B**) Relationship between neutrophil percentage in white blood cell (WBC) and serum Apo-A1 level in all enrolled subjects (*n* = 34). (**C**) Relationship between NLR and serum Apo-A1 level in all enrolled subjects. (**D**) Relationship between neutrophil percentage in WBC and serum Apo-A1 level in UTUC patients. The Spearman method was used for determination of correlation. * *p* < 0.05.

To identify the factors responsible for modulating neutrophil immunosuppression, we conducted proteomic arrays to assess the composition of supernatants. Among the 105 proteins analyzed, we identified 14 differential factors that exhibited a significant increase in tumor samples (fold change > 2 and *p* < 0.05, when compared to the control medium), including angiogenin, adiponectin, apolipoprotein A1 (Apo-A1), macrophage migration inhibitory factor (MIF), matrix metallopeptidase 9 (MMP-9), endoglin, lipocalin-2, trefoil factor 3 (TFF3), complement D, CD14, CD40L, cystatin C, hepatocyte growth factor (HGF), and thrombospondin-1 (Figure 2). In other studies, these factors have been found to exert direct influences on neutrophils, including the inhibition of degranulation (angiogenin, Apo-A1) [25,26], the suppression of apoptosis (adiponectin, MIF) [27,28], the promotion of neutrophil migration (CD40L) [29], and the enhancement of chemotaxis (thrombospondin-1) [30]. Among these factors, treatment with angiogenin or Apo-A1 inhibited the inflammatory responses of neutrophils, whereas other factors tended to contribute to neutrophil-mediated inflammation. However, factors involved in neutrophil activation, such as CCL20 and GM-CSF, were not significantly increased in UTUC. Of note, Apo-A1 was identified as a novel urinary biomarker for bladder cancer [31]. In addition, patients with hepatocellular carcinoma exhibited higher serum Apo-A1 levels compared with normal controls [32]. In the present study, UTUC patients exhibited elevated serum Apo-A1 expression compared with healthy controls (Figure 3A). Furthermore, increased serum Apo-A1 levels were correlated with higher neutrophil levels and elevated NLR values in the peripheral blood (Figure 3B,C). In UTUC patients, the percentage of neutrophils in peripheral white blood cells increased along with an elevated serum Apo-A1 level (Figure 3D). These findings link the increased neutrophils in blood to Apo-A1 expression, suggesting possible the modulation of neutrophils within the tumor microenvironment of UTUC.

### 3.2. Elevated Serum Apo-A1 Was Correlated with Tumor-Infiltrating Neutrophils and T Cells

**Figure 4:**
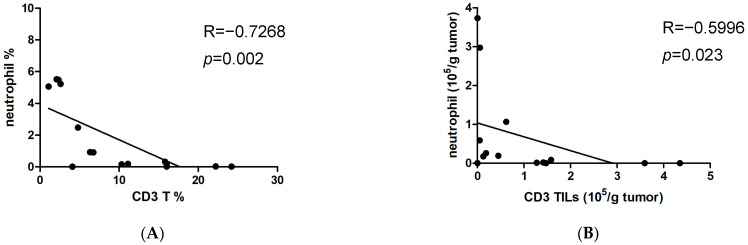

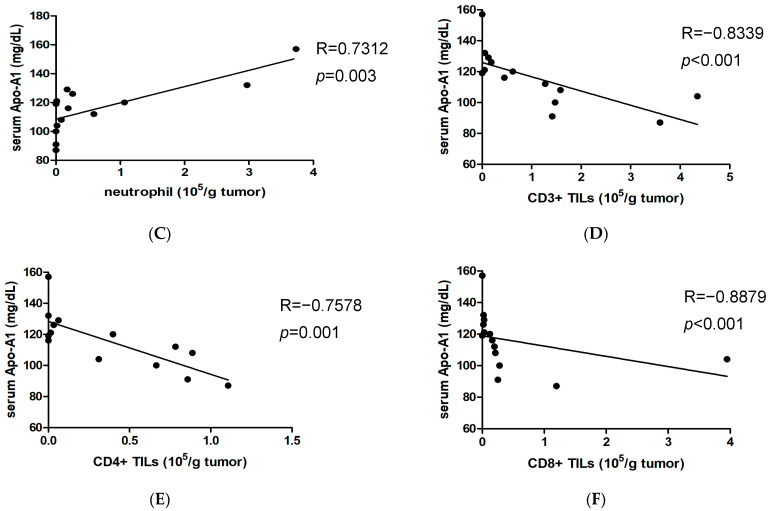
Serum Apo-A1 levels are negatively correlated with tumor-infiltrating T lymphocytes. Infiltrating cells isolated from tumor tissues of UTUC patients were counted and stained with fluorescent dye-conjugated Abs. Cell populations were then analyzed by flow cytometry. (**A**) The correlation between CD3 T cells (CD3^+^% in CD45) and neutrophils (CD66b^+^CD15^+^% in CD45) was determined. (**B**) The number of infiltrating CD3 TILs (CD45^+^CD3^+^) per gram of tumor was correlated with that of infiltrating neutrophils. (**C**) The number of infiltrating neutrophils per gram of tumor was correlated with the serum Apo-A1 level of UTUC patients. The numbers of infiltrating (**D**) CD3^+^ TILs, (**E**) CD4^+^ TILs (CD45^+^CD3^+^CD4^+^), or (**F**) CD8^+^ TILs (CD45^+^CD3^+^CD8^+^) per gram of tumor were correlated with the serum Apo-A1 levels of UTUC patients. The Spearman method was used for the determination of correlation.

Given that a higher NLR value reflects the relative depletion of lymphocytes [16], we examined UTUC tumor tissue for the presence of neutrophils and T lymphocytes. The results indicated a negative correlation between the percentage of tumor-infiltrating neutrophils and CD3^+^ TILs (Figure 4A). The absolute numbers of infiltrating neutrophils were also inversely associated with infiltrating CD3^+^ TILs (Figure 4B). This indicates that T cell deficiency in UTUC is linked to an increase in tumor-infiltrating neutrophils. Importantly, the absolute numbers of infiltrating neutrophils per gram of UTUC tumor showed a positive correlation with serum Apo-A1 levels (Figure 4C). Next, we determined whether elevated Apo-A1 expression in UTUC was associated with T cell depletion within the tumor. The absolute numbers of infiltrating CD3^+^ T cells per gram of UTUC tumor were negatively correlated with serum Apo-A1 levels (Figure 4D). Furthermore, both CD4^+^ and CD8^+^ T cell subsets showed similar results (Figure 4E,F). Taken together, these findings suggest that Apo-A1 has a propensity to modulate immunosuppression in UTUC, particularly by attenuating T cell proliferation.

## 4. Discussion

We examined the relationship between serum Apo-A1 levels and tumor-associated neutrophils and T lymphocytes in UTUC. Highly expressed Apo-A1 protein was found not only in tumor tissue, but also in the blood of UTUC patients. Among the subjects enrolled in this study, we observed an increase in NLR value concurrent with an elevated Apo-A1 level in the serum. In the UTUC tumor microenvironment, infiltrated neutrophils were negatively correlated with CD3^+^ T cells. In addition, the absolute numbers of infiltrating CD3^+^, CD4^+^, and CD8^+^ T cell populations within the UTUC tumor were inversely associated with serum Apo-A1 levels. These findings suggest that higher Apo-A1 levels portend increased neutrophils and a T cell-depleted milieu within the UTUC tumor microenvironment.

Apo-A1, a major structural protein of the high-density lipoprotein family, regulates cholesterol trafficking [33]. The multifunctional role of Apo-A1 in inflammation and cancer has been revealed [33]. For instance, Apo-A1 inhibits inflammation mediated by neutrophils or macrophages [34,35,36]. In the tumor microenvironment of ovarian cancer, Apo-A1-induced cholesterol efflux inhibits IFNγ-related gene expression, which in turn enhances IL-4 signaling in tumor-associated macrophages to promote immunosuppression [37]. Nevertheless, the prognostic role of Apo-A1 in various cancers remains controversial. Higher Apo-A1 levels were observed in the serum of patients with hepatocellular carcinoma and the tumor tissue of patients with small-cell lung carcinoma compared with normal controls [32]. In bladder cancer, Apo-A1 may be a diagnostic factor in which protein levels in the urine are increased along with cancer progression [31,38]. In contrast, the reduction in Apo-A1 levels in the serum is a poor prognostic factor in non-small-cell lung carcinoma, prostate cancer, and renal cell cancer [32]. Interestingly, Zeng et al. indicated that a high level of APOA1 mRNA in tumor tissue is correlated with worse overall and disease-free survival in renal clear cell carcinoma, whereas a low level of preoperative Apo-A1 protein in the serum is an unfavorable factor for overall survival [39]. Of note, a negative correlation between Apo-A1 expression and immune signature was evident, including CD8 T cells, macrophages, neutrophils, and dendritic cells, in tumor tissue [39]. The present study demonstrates that Apo-A1 protein levels are increased in both the tumor tissue and serum of patients with UTUC. Apo-A1 levels in the serum positively correlate with NLR or tumor-infiltrating neutrophils in patients with UTUC. Moreover, tumor-infiltrating T cell populations negatively correlate with serum Apo-A1 or tumor-infiltrating neutrophils. These findings indicate a role for Apo-A1 levels in UTUC and its correlation with neutrophils and T cells in the tumor microenvironment.

The oncologic outcomes of UTUC patients undergoing nephroureterectomy are impacted by treatment-, patient-, and tumor-related factors [40]. For example, a higher preoperative NLR is associated with poorer outcomes, whereas perioperative chemotherapy was shown to improve survival. Although the evidence and sample sizes of neoadjuvant immunotherapy in patients with UTUC are limited, some immunologic features, such as intratumoral CD8^+^ T cells or IFN-γ signatures, are considered indicators of responders administered immune checkpoint inhibitor therapy [41]. In the present study, infiltrating T lymphocytes within a UTUC tumor are inversely associated with neutrophils, providing evidence of the intratumoral immune signature of UTUC and the potential to improve personalized therapy.

This study has some limitations. Although we determined the absolute number and percentage of infiltrated cells in primary tumors, the number of enrolled patients may be insufficient, and a larger sample size may increase the significance of the results. Cheng et al. proposed that more stromal tumor-infiltrating lymphocytes, as defined by immunohistochemical staining, are associated with improved survival in UTUC [42]. In the present study, the results regarding tumor-infiltrating neutrophils and T lymphocytes were assessed using flow cytometry, which is relatively restricted by tumor size and the number of isolated cells. Therefore, the prognostic significance of these infiltrating cells or the serum Apo-A1 level in the present study was not determined. Until now, the diagnosis of UTUC using urinary or blood samples has seemed to lack effective and sufficient evidence due to limitations in the number of cases [43]. Therefore, the detection of blood Apo-A1 protein in a larger sample size may enhance the significance of Apo-A1 as a novel biomarker for UTUC.

## 5. Conclusions

We demonstrated that increased Apo-A1 levels are associated with increased neutrophils and decreased T lymphocytes in the tumor microenvironment of UTUC. This study provides novel insights into immunomodulation in UTUC and potential therapeutic targets for UTUC treatment.

## Figures and Tables

**Table 1:** Characteristics of the study participants. * *p* < 0.05, compared with healthy controls.

	UTUC Patients (*n* = 14)	Healthy Controls (*n* = 20)
Age, year (mean ± SD)	72.57 ± 11.02	67.75 ± 5.98
Gender, *n* (%)		
Male	5 (36)	11 (55)
Female	9 (64)	9 (45)
Neutrophils, %	* 72.99 ± 10.34	61.37 ± 5.85
Lymphocytes, %	* 19.16 ± 9.58	28.89 ± 5.21
NLR ratio	* 4.2 ± 3.72	2.23 ± 0.68
Tumor site, *n* (%)		
Ureter	10 (71)	
Renal pelvic	9 (64)	
Both	5 (36)	
T stage, *n* (%)		
≤pT2	6 (43)	
>pT2	8 (57)	

## Data Availability

Data are contained within the article.
